# Modified procedure for reconstructing the inferior wall of the orbit: identification of a reliable new landmark

**DOI:** 10.1007/s00405-022-07587-1

**Published:** 2022-08-11

**Authors:** Kosuke Takabayashi, Yohei Maeda, Nobuya Kataoka

**Affiliations:** 1 Department of Otorhinolaryngology, Japanese Red Cross Asahikawa Hospital, Asahikawa City, Hokkaido Japan; 2 Department of Otorhinolaryngology–Head and Neck Surgery, Osaka University Graduate School of Medicine, 2-2 Yamada-oka, Suita City, Osaka 565-0871 Japan; 3 Department of Otorhinolaryngology, Japan Community Health Care Organization Osaka Hospital, Osaka City, Osaka Japan; 4 Department of Ophthalmology, Japanese Red Cross Asahikawa Hospital, Asahikawa City, Hokkaido Japan

**Keywords:** Greater wing of the sphenoid bone, Inferior orbital fissure, Bone–mucosal flap, Transorbital approach, Transnasal approach

## Abstract

**Background:**

In orbital floor reconstruction, fractures involving the slope of the posterior end of the orbital floor make it difficult to determine the best location for implant placement. Therefore, landmarks for reconstruction are desirable to perform safe and reproducible reconstruction surgery.

**Methods:**

We developed a surgical procedure that focuses on three orbital landmarks: the infraorbital nerve, the inferior margin of the greater wing of the sphenoid bone, and the posterior superior wall of the maxilla.

**Conclusions:**

Landmark-based orbital floor fracture reconstruction enables accurate reconstruction of fractures that extend to the slope of the posterior end of the orbital floor.

**Supplementary Information:**

The online version contains supplementary material available at 10.1007/s00405-022-07587-1.

## Relevant surgical anatomy

**Fig. 1:**
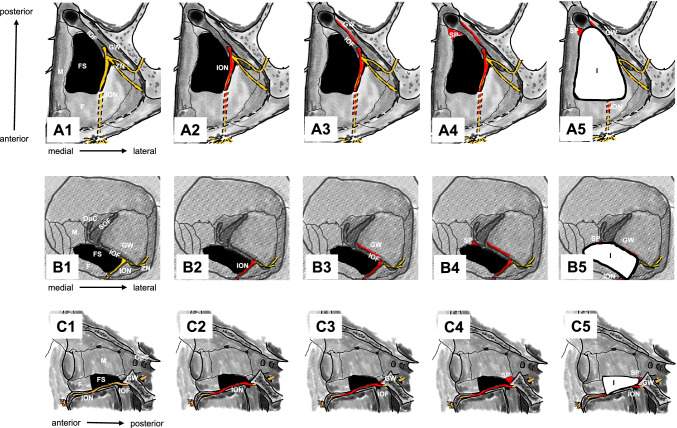
Stepwise schema of landmark identification during the procedure. Only bones and the maxillary nerve and its branches are shown so that the landmarks are better recognized. A1–5 show the left orbital floor from the cranial side. B1–5 show the left orbit from the front. C1–5 show the left orbit from the outside and going inward. The black area indicates a fracture site. The yellow line represents the maxillary nerve and its branches, the infraorbital nerve and the zygomatic nerve (A1, B1, C1). Identify the first landmark, the infraorbital nerve shown in red, and proceed posteriorly (A2, B2, C2). Incise the periosteum on the cranial side of the pterygopalatine fossa at the inferior orbital fissure and identify the second landmark, the inferior margin of the greater wing of the sphenoid bone, shown in red, and expose it to the inferomedial margin (A3, B3, C3). Identify the third landmark, the superior posterior wall of the maxillary sinus, shown in red, medial to the inferomedial margin of the greater wing of the sphenoid bone across the inferior orbital fissure (A4, B4, C4). Place the implant in contact with the third landmark in the direction of the orbital apex. The posterior aspect of the implant should be in contact with the second landmark. The outside of the implant should be in contact with the first landmark, with complete coverage of the blowout site. The white area surrounded by a black line indicates the implant (A5, B5, C5). *F* orbital floor, *FS* fracture site, *GW* greater wing of the sphenoid bone, *I* implant, *IOF* inferior orbital fissure, *ION* infraorbital nerve, *M* medial orbital wall, *OpC* optic canal, *SOF* superior orbital fissure, *SP* superior posterior wall of the maxillary sinus, *ZN* zygomatic nerve

Three landmarks are used to perform surgical manipulations safely and reproducibly in orbital floor reconstruction. The first is the infraorbital nerve, which indicates the height of the orbital floor, runs from the foramen rotundum to the cranial side of the pterygopalatine fossa, and then to the floor of the orbit [1] (Fig. 1A2, B2, C2). Proceeding posteriorly along the superior border of the infraorbital nerve, it dead-ends at the periosteum on the cranial side of the pterygopalatine fossa at the inferior orbital fissure. An incision in the periosteum along the inferior orbital fissure at the level of the superior border of the infraorbital nerve identifies the inferior margin of the greater wing of the sphenoid bone, which is the second landmark. It does not become displaced or fractured in fractures of the inferior wall of the orbit (Fig. 1A3, B3, C3). Advancing medially along the inferior margin of the greater wing of the sphenoid bone, while cutting the periosteum leads to the inferomedial end of the greater wing. The superior posterior wall of the maxillary sinus is the third landmark, because it does not become displaced or fractured in fractures of the inferior wall of the orbit [2]. It can be identified medially across the inferior orbital fissure at the inferomedial end of the greater wing of the sphenoid bone (Fig. 1A4, B4, C4).

## Description of the technique

**Fig. 2:**
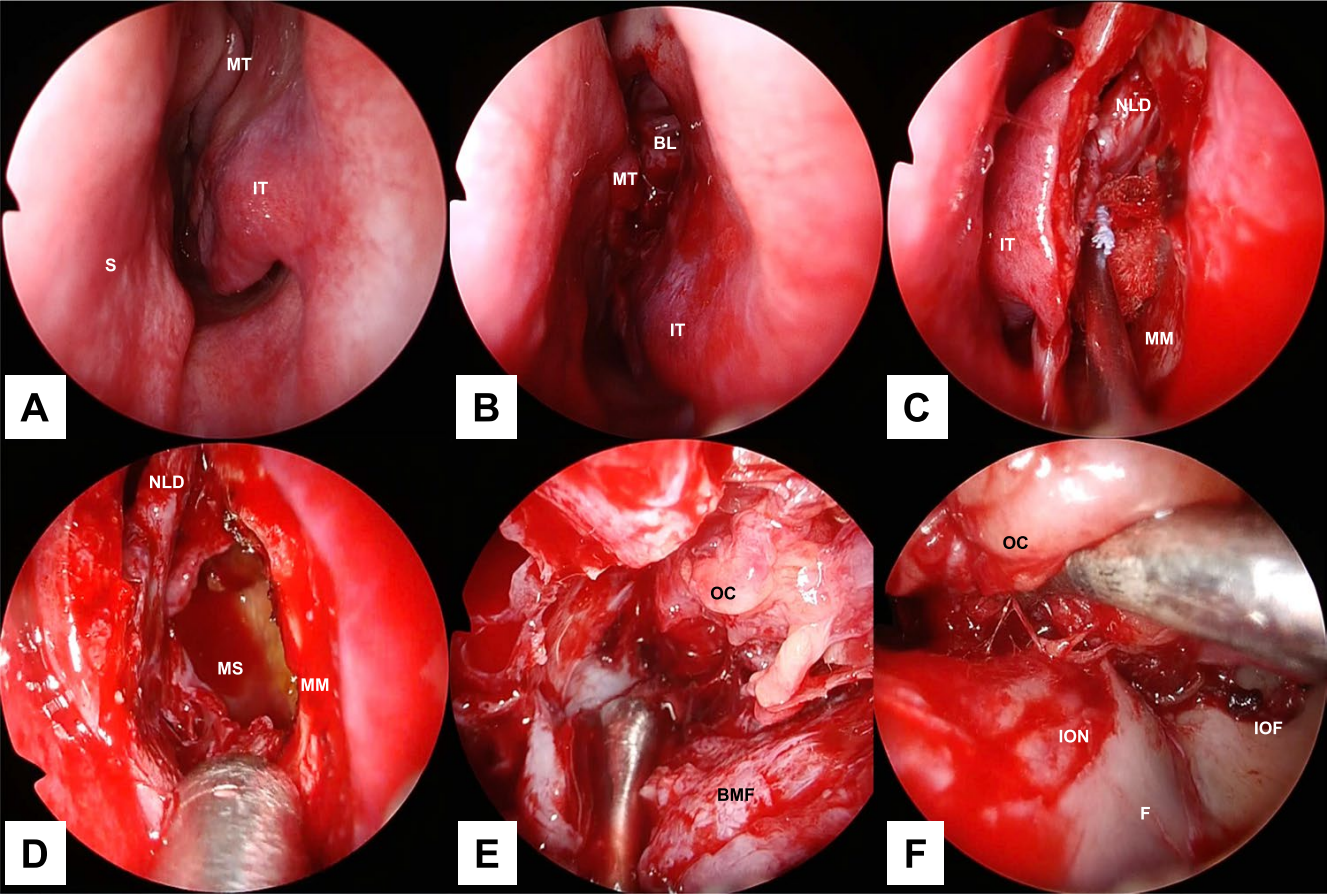
Stepwise endoscopic images of the procedure: the process for identifying the first landmark. Images **A**–**E** are views from the 0 degree telescope with the transnasal approach. Image **F** is a view from the 0 degree telescope with the transorbital approach. The left nasal cavity was shown (**A**). Anterior ethmoidectomy and maxillectomy were performed (**B**). A prelacrimal approach was performed to manipulate the maxillary sinus (**C**, **D**). The fractured bone fragments were elevated as a local bone–mucosal flap (**E**). The infraorbital nerve was identified (**F**). *BL* basal lamella of the middle turbinate, *BMF* bone–mucosal flap, *F* orbital floor, *IOF* inferior orbital fissure, *ION* infraorbital nerve, *IT* inferior turbinate, *MM* medial wall of the maxillary sinus, *MS* maxillary sinus, *MT* middle turbinate, *NLD* nasolacrimal duct, *OC* orbital contents, S nasal septum

**Fig. 3:**
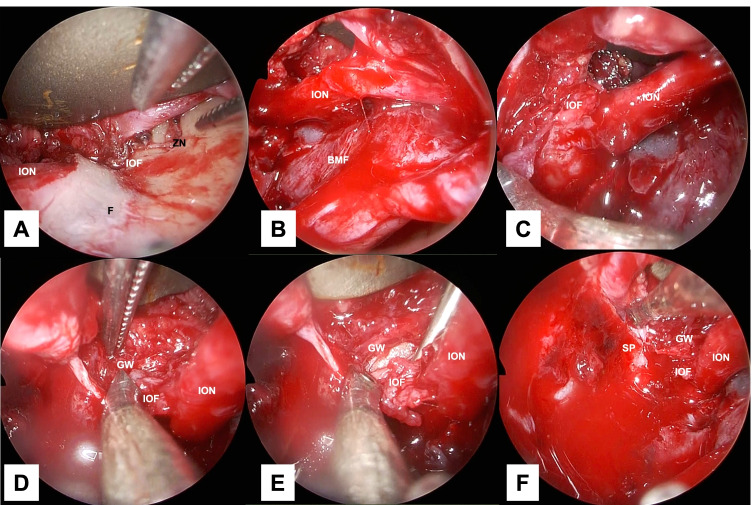
Stepwise endoscopic images of the procedure: identification of the first landmark to identification of the third landmark. Image **A** is a view from the 0 degree telescope with the transorbital approach. Images **B** and **C** are views from the 70 degree telescope with the transnasal approach. Images **D**–**F** are views from the 0 degree telescope with the transnasal approach. The zygomatic nerve is lateral to the infraorbital nerve (**A**). The infraorbital nerve was identified (**B**), and proceeding posteriorly along the infraorbital nerve, the cranial side of the pterygopalatine fossa was identified at the inferior orbital fissure (**C**). The periosteum was incised in the layer just above the infraorbital nerve. The inferior margin of the greater wing of the sphenoid bone was identified (**E**). Proceeding medially (**E**), the superior posterior wall of the maxillary sinus was found medial to the inferomedial margin of the greater wing of the sphenoid bone across the inferior orbital fissure (**F**). *BMF* bone–mucosal flap, *F* orbital floor, *GW* greater wing of the sphenoid bone, *IOF* inferior orbital fissure, *ION* infraorbital nerve, *SP* superior posterior wall of the maxillary sinus, *ZN* zygomatic nerve

**Fig. 4:**
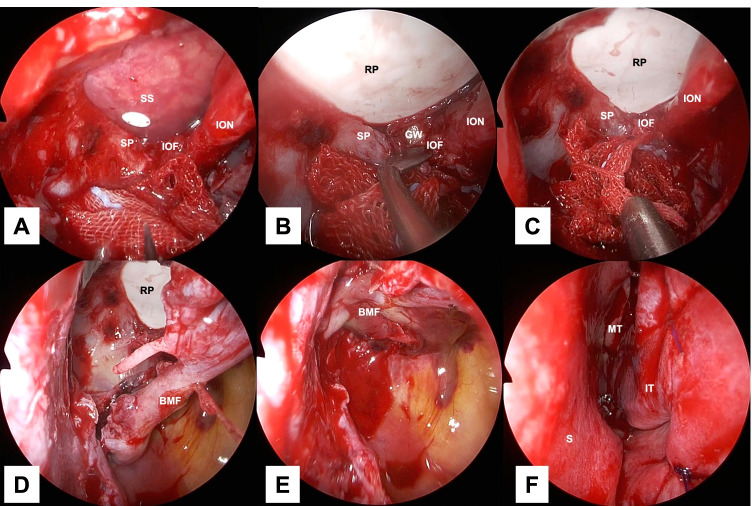
Stepwise endoscopic images of the procedure: reconstructing the orbital floor. Images **A**–**F** are views from the 0 degree telescope with the transnasal approach. The herniated tissue was placed in the orbit with a silicone silastic sheet (**A**). A rigid plate was implanted. However, there was a space between the inferior margin of the greater wing of the sphenoid bone and the plate and between the superior posterior wall of the maxillary sinus and the plate (**B**). The plate was reshaped to eliminate the spaces between the plate and the inferior margin of the greater wing of the sphenoid bone and the superior posterior wall of the maxillary sinus, respectively (**C**). The orbit and maxillary sinus were separated with a local bone–mucosal flap (**D**, **E**). The left nasal cavity at the end of the procedure was shown (**F**). *BMF* bone–mucosal flap, *GW* greater wing of the sphenoid bone, *IOF* inferior orbital fissure, *ION* infraorbital nerve, *IT* inferior turbinate, *MT* middle turbinate, *RP* rigid plate, *S* nasal septum, *SP* superior posterior wall of the maxillary sinus, *SS* silicone silastic sheet

**Fig. 5:**
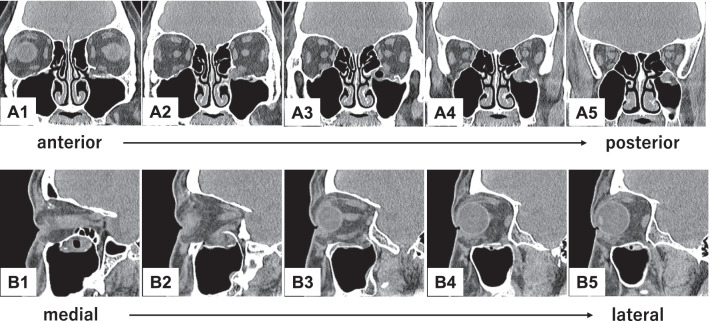
Preoperative computed tomography (CT) images. A1–5 are reformatted coronal CT images in anterior to posterior order. B1–5 are reformatted sagittal CT images in medial to lateral order. Images show an orbital floor fracture that extends to the slope of the posterior end of the orbital floor

**Fig. 6:**
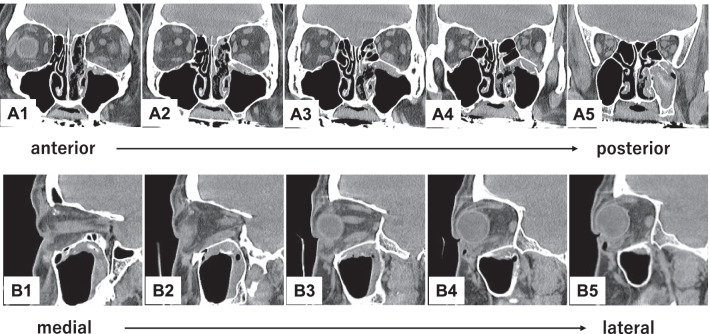
Postoperative computed tomography (CT) images. A1–5 are reformatted coronal CT images in anterior to posterior order. B1–5 are reformatted sagittal CT images in medial to lateral order. Images B3 and B4 show the implant inserted posterior to the inferior orbital fissure and in contact with the inferior margin of the greater wing of the sphenoid bone. The orbit and the maxillary sinus were completely separated by a bone–mucosal flap; the implant was not exposed to the maxillary sinus

The procedure combines an endoscopic transnasal approach with a transorbital approach via a subciliary incision. The surgeon using the transnasal approach manipulates the maxillary sinus via a prelacrimal approach (Fig. 2A–D). The fractured bone fragments are elevated into the maxillary sinus by detaching them from the orbital contents as a local bone–mucosal flap with a posterior pedicle, without detaching them from the maxillary mucosa (Fig. 2E). Next, the infraorbital nerve is identified (Figs. 1A2, B2, C2, 2F, 3B). The surgeon proceeds posteriorly along its superior border to identify the inferior orbital fissure (Fig. 3C). The periosteum is incised to identify the inferior margin of the greater wing of the sphenoid bone (Figs. 1A3, B3, C3, 3D). The surgeon then proceeds medially (Figs. 1A3, B3, C3, 3E) to identify the superior posterior wall of the maxillary sinus (Figs. 1A4, B4, C4, 3F). After the fractured bone fragments have been elevated as a bone–mucosal flap and the three landmarks (infraorbital nerve, line of the inferior margin of the greater wing of the sphenoid bone, and superior posterior wall of the maxillary sinus) have been identified, the herniated orbital contents are restored completely with a silicone silastic sheet (Eyeball restraint insert; Koken, Tokyo, Japan) (Fig. 4A). Next a poly-l-lactic acid/hydroxyapatite (PLLA/HA) sheet (Super Fixorb MX® 0.3 mm sheet; Teijin Medical Technologies, Osaka, Japan), which is a resolvable rigid plate, is molded and implanted transorbitally to cover the blowout site (Figs. 1A5, B5, C5, 4B, C). Next, the silastic sheet is removed after implantation of the PLLA/HA sheet. The sheet is placed so that its deepest part is on the superior posterior wall of the maxillary sinus and its posterior edge is in contact with the line of the inferior margin of the greater wing of the sphenoid bone. The sheet extends to the inferior margin of the greater wing of the sphenoid bone posterior to the posterior wall of the maxillary sinus, making it possible to completely cover the fracture site, even when the slope of the posterior end of the orbital floor is fractured. Finally, the orbit and maxillary sinus are separated completely by covering the fracture site with a local bone–mucosal flap (Fig. 4D, E). Preoperative computed tomography (CT) showed that the fracture site extended to the slope of the posterior end of the orbital floor (Fig. 5). Postoperative CT showed that the sheet was placed more posterior to the posterior wall of the maxillary sinus and was in contact with the inferior margin of the greater wing of the sphenoid bone (Fig. 6B3, B4). Furthermore, the plate was not exposed to the maxillary sinus, because the orbit was separated from the maxillary sinus by a local bone–mucosal flap (Fig. 6).

## Discussion

We developed the inferior margin of the greater wing of the sphenoid bone as a new landmark for orbital floor fracture reconstruction. There have been no previous reports focusing on this landmark, because the greater wing of the sphenoid bone is a part of the lateral orbital wall and not part of the inferior wall. In addition, the inferior wall of the orbit terminates posteriorly in the inferior orbital fissure. The greater wing of the sphenoid bone is always posterior to the periosteum on the cranial side of the pterygopalatine fossa at the inferior orbital fissure. Therefore, we incise the periosteum medial to the inferior orbital nerve in a layer with the superior border of the nerve. The zygomatic nerve emerges from the inferior orbital fissure into the orbit. Since the zygomatic groove in which the zygomatic nerve runs is located lateral to the infraorbital nerve, a medial incision is safe (Figs. 1A, B, 3A).

As a landmark, the inferior margin of the greater wing of the sphenoid bone has three advantages. First, the infraorbital nerve and the superior posterior wall of the maxillary sinus can be safely connected by proceeding along the new landmark, because the landmark runs along the inferior orbital fissure. The surgeon can follow the landmark without accidental entry into the intraconal space. Second, this new landmark can serve as an indicator of the height of the posterior margin of the orbital floor, even when the height to be reconstructed is difficult to identify due to a fracture extending to the slope of the posterior end of the orbital floor. This landmark enables highly reproducible surgery for posterior fractures, which have been reported to be difficult [3, 4]. Third, the implant can be placed in contact with the inferior margin of the greater wing of the sphenoid bone and posterior to the posterior edge of the inferior wall, enabling the implant to completely cover the fracture site even when the slope of the posterior end of the orbital floor is fractured.

We used the subciliary approach as part of the transorbital approach, with which we have technical familiarity. Alternatively, the transconjunctival approach can also be used. The transconjunctival approach requires more technical skill than the subciliary approach. Nevertheless, the risk of complications such as ectropion or scleral show is reduced than subciliary approach and a larger operative field can be achieved if necessary [5].

## Indications

This procedure is indicated for non-linear infraorbital wall fractures.

## Limitations

A limitation of this procedure is that there has not yet been follow-up until the resolution of the soluble implant. Further accumulation of cases and careful follow-up are required. This technique might not be indicated for extensive fractures of the infraorbital wall when there is no space to place an implant.

## How to avoid complications

Recognizing the three landmarks for this technique can help avoid damage to the contents of the orbit.

## Specific perioperative considerations

Immediately after surgery, check for the presence of optic neuropathy. Attention should be paid to aggravation of orbital swelling due to postoperative hemorrhage.

## Specific information to give to the patient about surgery and potential risks

Although there is a risk of residual ocular motility disturbance and postoperative eye socket depression, this procedure is the best technique for avoiding these complications.

## A summary of 10 key points

1. This procedure focuses on three orbital landmarks: the infraorbital nerve, the inferior margin of the greater wing of the sphenoid bone, and the posterior superior wall of the maxilla.
2. We emphasize the inferior margin of the greater wing of the sphenoid bone as a new landmark.
3. The infraorbital nerve is the first landmark for safely identifying the inferior orbital fissure.
4. The second landmark, the inferior margin of the greater wing of the sphenoid bone, can be identified after incising the periosteum of the inferior orbital fissure.
5. The inferior margin of the greater wing of the sphenoid bone helps the surgeon safely identify the superior posterior wall of the maxillary sinus as the third landmark.
6. The third landmark, the superior posterior wall of the maxillary sinus, can be identified medially across the inferior orbital fissure at the inferomedial end of the greater wing of the sphenoid bone.
7. The third landmark does not become displaced or fractured in fractures of the inferior wall of the orbit.
8. A resolvable rigid plate is implanted transorbitally so that it covers the fracture site and makes contact with all landmarks.
9. The combined endoscopic transnasal and transorbital approach improves the safety and accuracy of the operation.
10. Re-separation of the orbit and the maxillary sinus with a local bone–mucosal flap avoids exposure of the implant to the sinus and reduces the risk of infection.

## Supplementary Information

Below is the link to the electronic supplementary material.

Supplementary file1 (MP4 92875 KB)
